# Characterization of a Lytic Bacteriophage vB_SurP-PSU3 Infecting *Staphylococcus ureilyticus* and Its Efficacy Against Biofilm

**DOI:** 10.3389/fmicb.2022.925866

**Published:** 2022-07-18

**Authors:** Hyemin Kwon, Seon Young Park, Min-Soo Kim, Sang Guen Kim, Se Chang Park, Ji Hyung Kim

**Affiliations:** ^1^Department of Microbiology and Molecular Biology, College of Bioscience and Biotechnology, Chungnam National University, Daejeon, South Korea; ^2^Infectious Disease Research Center, Korea Research Institute of Bioscience and Biotechnology, Daejeon, South Korea; ^3^Division of Animal and Dairy Sciences, College of Agriculture and Life Science, Chungnam National University, Daejeon, South Korea; ^4^Laboratory of Aquatic Biomedicine, College of Veterinary Medicine and Research Institute for Veterinary Science, Seoul National University, Seoul, South Korea; ^5^Department of Food Science and Biotechnology, College of BioNano Technology, Gachon University, Seongnam, South Korea

**Keywords:** *Staphylococcus ureilyticus*, biofilm, bacteriophage, *Andhravirus*, bio-control

## Abstract

In response to the increasing nosocomial infections caused by antimicrobial-resistant coagulase-negative staphylococci (CoNS), bacteriophages (phages) have emerged as an alternative to antibiotics. *Staphylococcus ureilyticus*, one of the representative species of the CoNS, is now considered a notable pathogen that causes nosocomial bloodstream infections, and its biofilm-forming ability increases pathogenicity and resistance to antimicrobial agents. In this study, a lytic phage infecting *S. ureilyticus* was newly isolated from wastewater collected from a sewage treatment plant and its biological and antimicrobial characteristics are described. The isolated phage, named vB_SurP-PSU3, was morphologically similar to *Podoviridae* and could simultaneously lyse some *S. warneri* strains used in this study. The sequenced genome of the phage consisted of linear dsDNA with 18,146 bp and genome-based phylogeny revealed that vB_SurP-PSU3 belonged to the genus *Andhravirus*. Although its overall genomic arrangement and contents were similar to those of other members of the *Andhravirus*, the predicted endolysin of vB_SurP-PSU3 distinctly differed from the other members of the genus. The bacteriolytic activity of vB_SurP-PSU3 was evaluated using *S. ureilyticus* ATCC 49330, and the phage could efficiently inhibit the planktonic growth of the bacteria. Moreover, the anti-biofilm analysis showed that vB_SurP-PSU3 could prevent the formation of bacterial biofilm and degrade the mature biofilm *in vitro*. In an additional cytotoxicity assay of vB_SurP-PSU3, no significant adverse effects were observed on the tested cell. Based on these findings, the newly isolated phage vB_SurP-PSU3 could be classified as a new member of *Andhravirus* and could be considered an alternative potential biocontrol agent against *S. ureilyticus* infections and its biofilm.

## Introduction

*Staphylococcus ureilyticus*, also known as *S. cohnii* subsp. *urealyticus*, is a gram-positive coagulase-negative staphylococci (CoNS) (Kloos and Wolfshohl, 1991; Madhaiyan et al., 2020). *S. ureilyticus* has been considered a non-pathogenic animal and human commensal bacterium in past decades. However, as the use of indwelling or implanted foreign bodies is increasing owing to medical developments, *S. ureilyticus* has become a common causative agent of nosocomial infections; the representative disease of *S. ureilyticus* infection is bloodstream infection (BSI) (Petinaki et al., 2001; Jain et al., 2011; Singh et al., 2016). BSI has a significant morbidity and mortality rate, and is one of the most serious healthcare-associated infections (Vallés et al., 2008; McNamara et al., 2018). In nosocomial infections, the biofilm-forming ability of pathogens is a critical pathogenic factor aiding disease occurrence (Vestby et al., 2020). Biofilms are microbial communities with a three-dimensional extracellular matrix that can protect them from the external environment, including antimicrobial agents and the host immune system (Mah and O'Toole, 2001; Watters et al., 2016). Moreover, several studies have demonstrated that biofilms can adhere to the abiotic surfaces of medical devices and allow bacterial cells to survive there, and they therefore represent clinically important pathogenic factors in nosocomial infections (Singhai et al., 2012; Jamal et al., 2018). Although the biofilm of *S. ureilyticus* has rarely been studied, CoNS are reportedly known as biofilm-forming bacteria, and the pathogenesis of CoNS is closely related to the formation of biofilms (Szczuka et al., 2016; França et al., 2021). For these reasons, an adequate method to prevent and cure such *S. ureilyticus* infection is highly necessary.

Antibiotics are universal antimicrobial agents that can be used to remove a wide spectrum of pathogens. However, with the increasing usage of antibiotics, antimicrobial resistant (AMR) pathogens have emerged as a worldwide obstacle to treatment (Prestinaci et al., 2015). Similarly, several studies in human medicine have reported the global emergence of multidrug-resistant CoNS, including *S. ureilyticus* (Cuevas et al., 2004; Szewczyk et al., 2004; Gatermann et al., 2007; Koksal et al., 2009; Song et al., 2017). As a result, current antimicrobial agents are insufficient, and developing new alternatives to antibiotics is essential to control *S. ureilyticus*. Bacteriophages (phages), viruses that infect bacteria as hosts, and viruses with lytic cycles have recently been considered as promising alternatives to control pathogenic AMR bacteria such as *Pseudomonas aeruginosa, Escherichia coli, Klebsiella pneumoniae, Salmonella enterica, Enterococcus* spp. (including VRE), *Streptococcus pneumonia*, and *Staphylococcus* spp. (Jado et al., 2003; El Haddad et al., 2019; Kuptsov et al., 2020). Furthermore, several recent studies have suggested that the potential of Staphylococcal phages could be used in other applications (including degradation of biofilms) beyond controlling AMR bacteria (Li et al., 2021; Zhang et al., 2021; Shahin et al., 2022). Until recently, phages infecting *S. aureus* have been one of the most thoroughly investigated types owing to the high rate of AMRs (including MRSA) (Salas et al., 2020; Walsh et al., 2021). As nosocomial infections by CoNS increase, studies on the efficacy of phages infecting CoNS and bacterial biofilms have been conducted (Doub et al., 2021; Valente et al., 2021). However, despite their potential clinical relevance, the phages infecting *S. ureilyticus* are relatively scarce compared to those for other species.

This study reports on a lytic phage infecting *S. ureilyticus* with a strong potential for biotechnological applications. The biological and genomic characteristics of the phage were examined as well as its ability to eradicate the biofilm of *S. ureilyticus*. This is the first report including a detailed characterization of phage infecting *S. ureilyticus*.

## Materials and Methods

### Bacterial Strains and Culture Condition (Wet Lab)

We used *S. ureilyticus* (ATCC 49330) to isolate the phage. The bacterial strains in this study included *S. aureus* (ATCC 12598, ATCC 6538, ATCC 29213, CCARM 3798, CCARM 3832, CCARM 3915, CCARM 3A170, and CCARM 3A200), *S. epidermidis* (ATCC 12228 and CCARM 3A794), *S. haemolyticus* (ATCC 29970 and CCARM 3A638), *S. warneri* (ATCC 27836, S1-4-2, S18-S-3, H6-3, and H71-3-2), *S. xylosus* (ATCC 29971), *S. saprophyticus* (ATCC 15305), and *Mammalicoccus sciuri* (SNUDS-18). The bacterial strains were cultured overnight in a tryptic soy broth (TSB; Difco, USA) with shaking, or on tryptic soy agar (TSA; Difco, USA) plates at 37°C. TSA soft agar overlays (0.7% agar) were used for further experiments, including phage isolation, propagation, and plaque count assays. The bacterial strains were stored at −80°C in TSB with 10% glycerol. Moreover, all the sections in Materials and methods were described by dividing them into “Wet lab” and “Dry lab” for the improvement of readability (Ranjbar et al., 2017).

### Phage Isolation (Wet Lab)

To isolate the phage infecting *S. ureilyticus*, environmental water samples were collected from a sewage treatment plant in Daejeon (Republic of Korea) using *S. ureilyticus* strain ATCC 49330. Phage isolation and propagation were performed using the double-layer agar method as previously described (Melo et al., 2014; Kim et al., 2019). Briefly, 1 ml of *S. ureilyticus* suspension was inoculated into the mixture of collected wastewater and TSB. Following overnight incubation at 37°C, the mixture was centrifuged at 10,000 × *g* for 20 min to remove the bacterial pellet, and the supernatant was filtered through a 0.22-μm membrane filter (Millipore, USA) to secure phages from the mixture. The filtrate was used to detect the presence of phages by confirming the formation of plaques on bacterial lawns. Following overnight incubation, plaque picking was repeated at least three times until a single plaque morphology was observed. For phage propagation, isolated phage suspension was incubated overnight with host bacteria by double-layer agar method, and the top-agar layer was collected, centrifuged at 10,000 × *g* for 10 min, and filtered. The filtered phage lysates were stored with 15% glycerol at −80°C.

### Transmission Electron Microscopy (Wet Lab)

The purified phages were deposited on glow-discharged carbon-copper grids and negatively stained with 2% (w/v) uranyl acetate (Electron Microscopy Sciences, Inc., USA). The stained phages were observed using transmission electron microscopy (TEM, JEM-1400 Plus; JEOL Ltd., Japan) at 120 kV at the Korea Basic Science Institute (Ochang, Korea).

### Host Range Determination (Wet Lab)

The host range of the isolated phage was determined against 22 strains of *Staphylococcus* spp. using spot assay as previously described (Kim et al., 2019). Briefly, 10 μl of phage lysate (~10^8^ PFU/ml) was spotted onto each bacterial overlay on TSA. Following overnight incubation at 37°C, the lytic ability of phages was evaluated based on the presence of plaques and the clearness of the lysis zones, which were assessed as clear (++), turbid (+), or no lysis (–) (Table 1).

**Table 1:** Host range of phage vB_SurP-PSU3.

**Bacterial species**			**Sensitivity**	**Source or reference**
Coagulase negative	*Staphylococcus ureilyticus*	ATCC 49330	++	ATCC
	*Staphylococcus warneri*	ATCC 27836	++	ATCC
		S1-4-2	–	Isolate^*^
		S18-S-3	–	Isolate^*^
		H6-3	+	Isolate^*^
		H71-3-2	–	Isolate^*^
	*Staphylococcus epidermidis*	ATCC 12228	–	ATCC
		CCARM 3A794	–	CCARM
	*Staphylococcus haemolyticus*	ATCC 29970	–	ATCC
		CCARM 3A638	–	CCARM
	*Staphylococcus xylosus*	ATCC 29971	–	ATCC
	*Staphylococcus saprophyticus*	ATCC 15305	–	ATCC
	Mammaliicoccus sciuri^*^	SNUDS-18	–	(Han et al., 2017)
Coagulase positive	*Staphylococcus aureus*	ATCC 12598	–	ATCC
		ATCC 29213	–	ATCC
		ATCC 6538	–	ATCC
		CCARM 3A170	–	CCARM
		CCARM 3A200	–	CCARM
		CCARM 3798	–	CCARM
		CCARM 3832	–	CCARM
		CCARM 3915	–	CCARM

*++, complete lysis; +, turbid lysis; –, no lysis*.

* *Isolated from companion animals and identified based on MALDI-TOF*.

** *Reclassification*.

### Thermal and pH Stability (Wet Lab)

Thermal and pH stability tests of the isolated phage were performed according to a previous protocol (Shahin et al., 2021). To examine the thermal stability of the phage, 1 ml phage suspension was incubated at 4, 25, 37, 56, and 80°C. To determine pH stability, 1 ml phage suspension mixed with different pH solutions (pH 3, 5, 7, 9, and 11) was incubated at 4°C. Each tube containing the phage suspension was incubated for 3 h and the phage titer was calculated using 10-fold serial dilution with the double-layer agar method.

### One-Step Growth Curve (Wet Lab)

To examine the growth characteristics of the isolated phage, its adsorption rate and burst size were measured as previously described (Kim et al., 2019). To calculate the adsorption rate within the incubation period, the phage suspension was mixed with the exponential growth phase of the host strain (~10^7^ CFU/ml) at a multiplicity of infection (MOI) of 0.001. The mixture was incubated at 37°C with shaking, and 100 μl of the mixture was taken every 5 min. The mixture was centrifuged at 12,000 × g for 3 min to precipitate the bacterial cells and obtain only the un-adsorbed free phage in the supernatant. Following centrifugation, 100 μl of the supernatant was serially diluted, and the adsorption rate of phage to the host bacteria over time was calculated. To estimate the number of progenies from a phage-infected bacterial cell, the phage suspension was mixed with the exponential growth phase of the host strain (~10^7^ CFU/ml) at an MOI of 0.001. Following shaking incubation at 37°C for 25 min, when the adsorption rate of phage was over 90%, the sample was centrifuged at 12,000 × g for 5 min, and the supernatant was discarded. The bacterial pellet was resuspended in pre-heated TSB and incubated at 37°C with shaking. A 100 μl portion of the mixture was taken for a total of 80 min, and the derived mixtures were used to calculate the burst size of the phage and identify a one-step growth curve.

### Bacteriolytic Activity (Wet Lab)

The bacteriolytic activity of the isolated phage was estimated as previously described (Shahin et al., 2019). The phage suspension was inoculated in the exponential growth phase of the host strain (~10^7^ CFU/ml) at MOI of 0.001, 0.01, 0.1, 1, 10, and 100, and TSB inoculated solely with *S. ureilyticus* was used as a positive control. The mixtures were incubated with shaking at 130 rpm for 10 h. A 1 ml portion of each mixture was taken at 2 h intervals and the bacterial density was measured at an absorbance of 600 nm using a UV/Vis spectrophotometer (K-Lab Co., Ltd., Korea).

### Phage DNA Extraction (Wet Lab)

To perform genome sequencing, phage genomic DNA was extracted based on a previous study with a slight modification (Kim et al., 2019). Briefly, DNase I (10 U/μl) and RNase A (10 U/μl) were added to the phage lysate and incubated at 37°C for 2 h. EDTA (0.5 M) and proteinase K (20 mg/ml) were mixed with the samples and incubated at 56°C for 3 h. AL buffer (QIAGEN Inc., USA) was added, and the mixture was incubated at 70°C for 15 min. The phage DNA was then purified using the phenol-chloroform extraction method (Sambrook and Russell, 2006).

### Genome Sequencing and Bioinformatic Analysis (Dry Lab)

The TruSeq DNA Nano Sample Preparation Kit (Illumina, Inc., USA) was used to prepare the sequencing libraries based on the manufacturer's instructions. The phage genome was sequenced using the Illumina HiSeq platform (Illumina, Inc., USA) at 211x coverage. *De novo* assembly was performed using SPAdes v3.13.0 software (Bankevich et al., 2012) and the genome was analyzed using PROKKA v1.14.6 (Seemann, 2014). The predicted function of open reading frames (ORFs) was annotated *via* Rapid Annotation using Subsystem Technology v2.0 (RAST) (Aziz et al., 2008), and the putative function of each ORF was predicted by the BlastP tool of the National Center for Biotechnology Information (NCBI). The conserved domain of each ORF was searched for *via* HHpred (https://toolkit.tuebingen.mpg.de/tools/hhpred) using the Pfam-A_v34 database, with an E-value threshold of <1.0. The number of transmembrane domains was confirmed using TMHMM v2.0 (https://services.healthtech.dtu.dk/service.php?TMHMM-2.0) and the molecular weight (Mw) and isoelectric point (pI) of the protein were estimated using ExPASy Compute pI/Mw (Wilkins et al., 1999). The presence of tRNA genes was confirmed using tRNAscan-SE v2.0 (Lowe and Eddy, 1997). The circular genome was visualized using Geneious Prime v.2021.1.1 (https://www.geneious.com). The data for several phage genomes closely related to the isolated phage were obtained from the GenBank database based on BlastN. EasyFig was used to compare the isolated phage and intimate phage genomes (Sullivan et al., 2011). Phylogenetic trees of phage conserved genes were aligned and constructed using MEGA X (Kumar et al., 2018) *via* the neighbor-joining method with 1000 bootstrap replications. The whole-genome phylogenetic tree was inferred using the Genome-BLAST Distance Phylogeny method (GBDP) in the Virus Classification and Tree Building Online Resource (VICTOR) (Meier-Kolthoff and Göker, 2017), and the tree was visualized using MEGA X.

### Anti-biofilm Activity (Wet Lab)

To evaluate the effectiveness of the phage on bacterial biofilm, the anti-biofilm analysis was performed as previously described (Kim et al., 2021). Briefly, for biofilm prevention assay, overnight *S. ureilyticus* was inoculated at 1% into fresh TSB and mixed with different MOIs of phage suspension (MOI of 0.001, 0.01, 0.1, 1, 10, and 100). Sterile phosphate-buffered saline (PBS) solution (Thermo Fisher Scientific Inc., USA) was used as a control instead of phage suspension. The mixtures were distributed into a 96-well polystyrene plate and incubated at 37°C for 24 and 48 h. Following incubation, the supernatant was removed from each well and the wells were washed twice to allow only biofilm to remain. The biofilm was stained with 0.1% crystal violet (CV) and dissolved in 95% ethanol. To quantify the total biofilm biomass, the optical density (OD) was measured using a plate reader (Molecular Devices Corp., USA) at a wavelength of 570 nm (*n* = 3). For biofilm degradation assay, *S. ureilyticus* was inoculated at 1% into fresh TSB in a 96-well polystyrene plate and incubated at 37°C for 24 h without shaking. The supernatant was then removed to retain the biofilm, and different MOIs of phage suspension (MOI of 0.1, 1, 10, 100, 1,000, and 10,000) and PBS (control) were distributed in each well. After 24 and 48 h of incubation at 37°C, the supernatant was removed and washed, and the remaining biofilm was stained with 0.1% CV and dissolved in 95% ethanol. Absorbance was measured using a plate reader at 570 nm to quantify the total biofilm biomass (*n* = 3).

### Confocal Laser Scanning Microscopy (CLSM) (Wet Lab)

The LIVE/DEAD™ BacLight™ Bacterial Viability Kit (Invitrogen™, Molecular Probes, USA) was used to distinguish live bacteria in the biofilm, and live bacteria were visualized using a confocal laser microscope (CLSM, LSM800, Carl Zeiss, Jena, Germany). Utilizing the manufacturer's protocol, the bacterial cultures (for the prevention assay) or biofilm (for the degradation assay) were cultured on coverslips (Paul Marienfeld GmbH & Co., Germany) for 24 and 48 h, respectively, at 37°C with different phage titers in a 6-well polystyrene plate and stained with SYTO 9 green-fluorescent nucleic acid stain (Invitrogen™, Molecular Probes, USA) for 20 min. The remaining stain was then washed with filtered distilled water, the coverslips in each well were moved onto a glass slide (Paul Marienfeld GmbH & Co., Germany), and the stained live *S. ureilyticus* was observed under CLSM.

### Cytotoxicity Assay (Wet Lab)

To confirm the cell toxicity of the phage, cytotoxicity assay was slightly modified as described previously (Porayath et al., 2018). A Quanti-Max WST-8 Cell Viability Assay kit (Biomax Ltd., Korea) was used to measure the number of living cells according to the manufacturer's protocol. African green monkey kidney (VERO-*CCl-81*) cells (10^5^ cells/well) were grown at 37°C and 5% CO_2_ in a 96-well polystyrene plate filled with RPMI1640 medium (Corning Inc., USA), supplemented with 10% fetal bovine serum (HyClone Laboratories Inc., USA) and 1% antibiotic/antimycotic solution (Thermo Fisher Scientific Inc., USA). Following overnight incubation, cells were washed twice, and the phage lysate (10^5^ PFU/ml) and heat-inactivated phage lysate were inoculated into each well (*n* = 3). Shiga toxin (Stx1) was used as a positive control. The mixtures were incubated for 3 h and 24 h at 37°C with 5% CO_2_. Quanti-Max™ was distributed in each well to quantify the cell viability, and the OD was measured at 450 nm.

### Statistical Analysis (Dry Lab)

A one-way analysis of variance (ANOVA) was conducted using the XLSTAT software in Excel (Office 365; Microsoft Corp., USA). The value of *P* < 0.05 was considered statistically significant.

### Culture Deposition and Nucleotide Sequence Accession Numbers

The *S. ureilyticus* phage was deposited in the Korean Collection for Type Cultures (KCTC) under KCTC 14773BP, and the complete genome sequence was deposited in the GenBank database under accession number OK574338.

## Results

### Isolation and Host Range of Phage vB_SurP-PSU3

**Figure 1:**
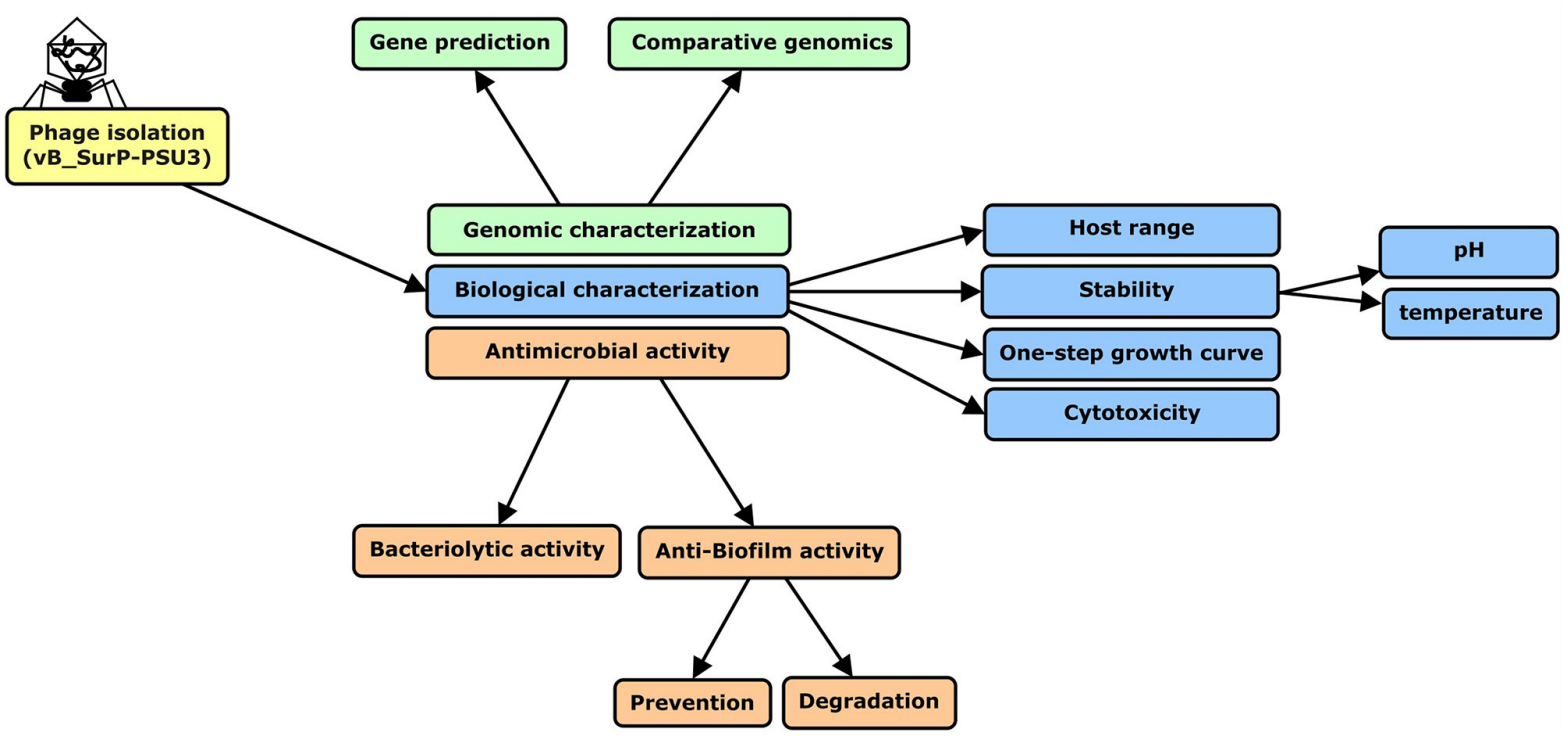
Flow chart of research process used in this study. *S. ureilyticus* ATCC49330 was used as host bacteria to isolate phage from wastewater. Genomic and biological characteristics and antimicrobial activity of isolated phage vB_SurP-PSU3 were measured.

**Figure 2:**
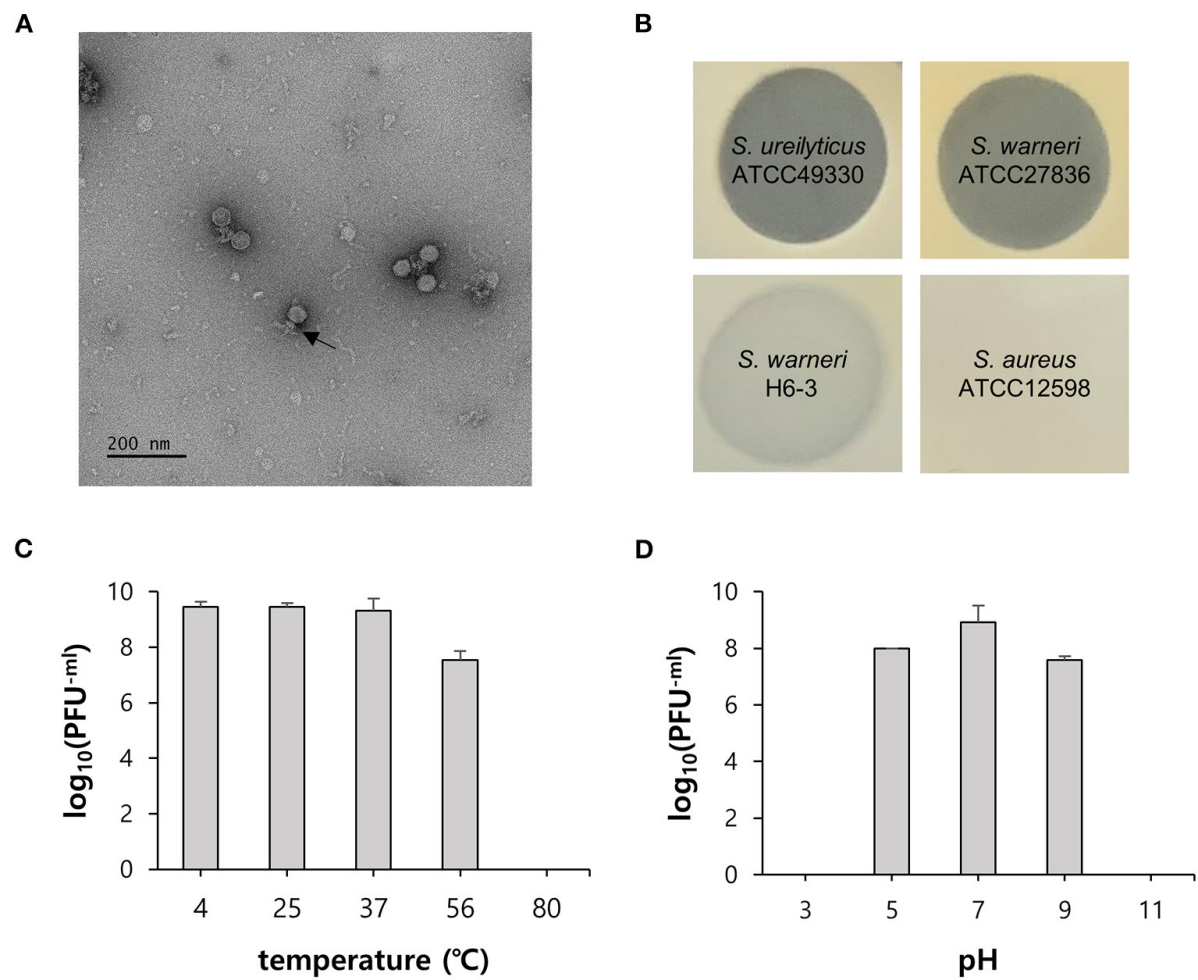
General characteristics of phage vB_SurP-PSU3. **(A)** Transmission electron micrograph. Scale bar = 200 nm. The arrow represents the phage tail. **(B)** Lytic characteristics of phage evaluated by spot assay on the different bacterial lawns. Representative examples: clear, turbid, and no lysis. **(C)** Thermal stability and **(D)** pH stability. The data **(C,D)** represent the mean ± SD (standard deviation) of three independent experiments.

The research process was summarized in a flow chart using CmapTools (Figure 1) (Cañas et al., 2004; Behzadi and Gajdács, 2021). The newly isolated *S. ureilyticus* phage, vB_SurP-PSU3, was isolated from wastewater and propagated with *S. ureilyticus* ATCC 49330 as the host. The host range of vB_SurP-PSU3 was tested against the 21 *Staphylococcus* strains used in this study. vB_SurP-PSU3 infected and showed a clear lytic plaque on *S. ureilyticus* ATCC 49330 and *S. warneri* ATCC 27836, and turbid plaque was formed in *S. warneri* H6-3; however, other staphylococcal strains did not show any plaques (Table 1 and Figure 2B).

### Biological Characteristics of Phage vB_SurP-PSU3

**Figure 3:**
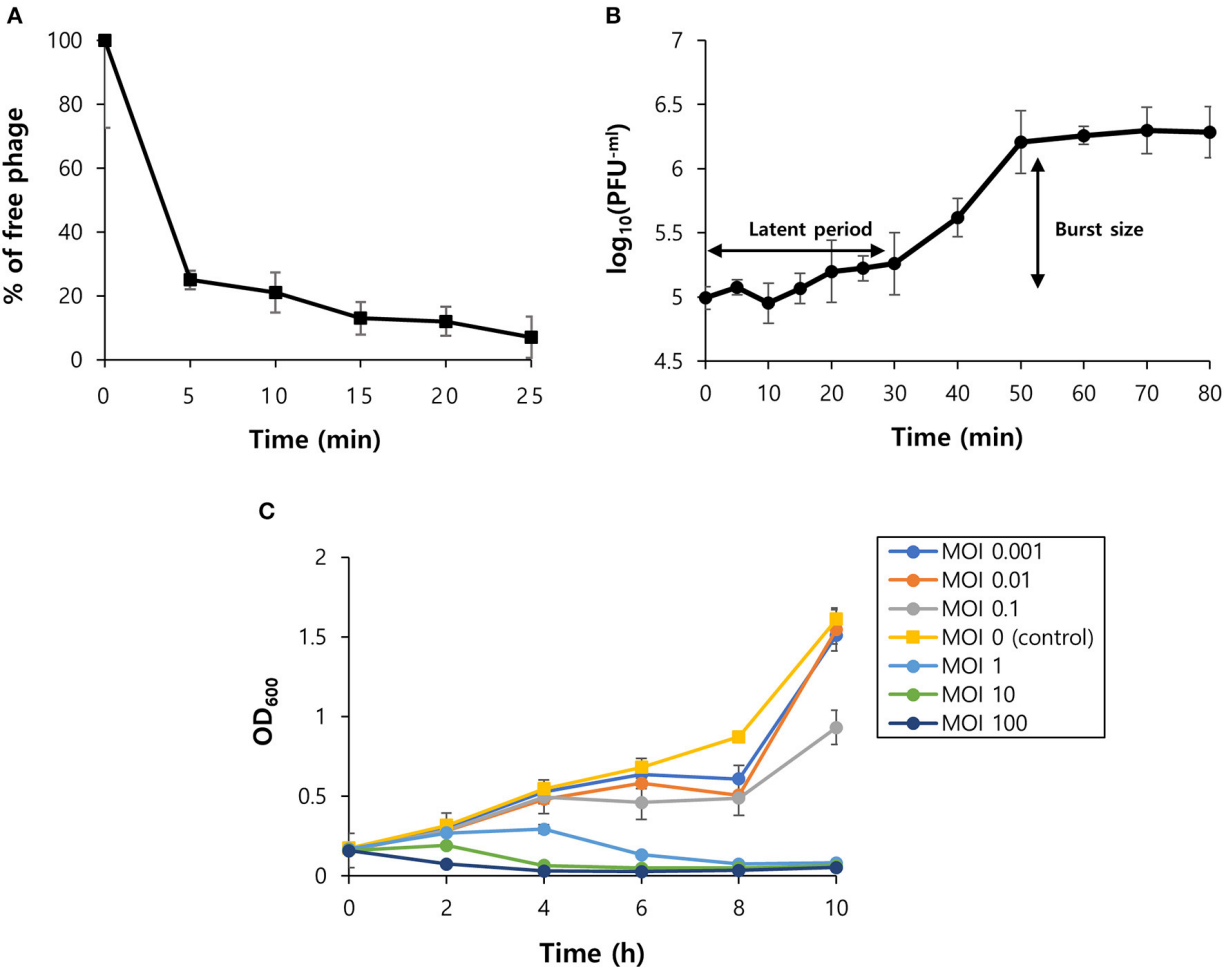
Biological characteristics of phage vB_SurP-PSU3. **(A)** The percentage of adsorbed phage particles on host surfaces at an MOI of 0.001. **(B)** One-step growth curve of phage infecting the host strain at an MOI of 0.001. **(C)** Bacteriolytic activity in the exponential phase of planktonic bacterial cells from MOIs of 0.001–100. The data **(A–C)** represent the mean ± SD of three independent experiments.

The virion morphology of phage vB_SurP-PSU3 was observed *via* TEM (Figure 2A). The phage vB_SurP-PSU3 has an icosahedral head (<50 nm in diameter) with a short, non-contractile tail, and its morphology is similar to the *Podoviridae* family. Under diverse thermal conditions, the phage was stable at temperatures ranging from 4 to 37°C (Figure 2C). At 56°C, the titer of phage vB_SurP-PSU3 decreased, but a high viability of > 10^7^ PFU/ml was maintained. However, plaques were not observed on the bacterial lawns at 80°C. Under diverse pH levels, phage vB_SurP-PSU3 had the highest survival rate at pH 7, and the phage titer decreased when the pH level was low or high, such as at pH 5 or 9 (Figure 2D). No viable phages were observed at pH 3 or 11, which represent extreme pH conditions. To estimate the life cycle and burst size of phage vB_SurP-PSU3, a one-step growth curve analysis was conducted at an MOI of 0.001. The adsorption affinity of the phage for bacteria was observed in advance, and it was over 90% after 25 min of inoculation (Figure 3A). Based on the adsorption affinity, the one-step growth curve showed that the latent period of the phage was 30 min, and the burst size was 148.5 virions per infected cell (Figure 3B).

### Planktonic Bacteriolytic Activity of Phage vB_SurP-PSU3

OD_600_ was measured every 2 h for 10 h to evaluate the effect of the phage titer on planktonic bacterial cells (Figure 3C). At low MOIs (0.001, 0.01, 0.1), the OD_600_ value decreased compared to the control group, but bacterial regrowth was observed after 8 h. This revealed that a low MOI could not inhibit bacterial regrowth over time. At high MOIs (1, 10, 100), the phage successfully lysed planktonic bacteria as the MOI value increased and continuously inhibited bacterial growth.

### Genomic Analysis of Phage vB_SurP-PSU3

**Figure 4:**
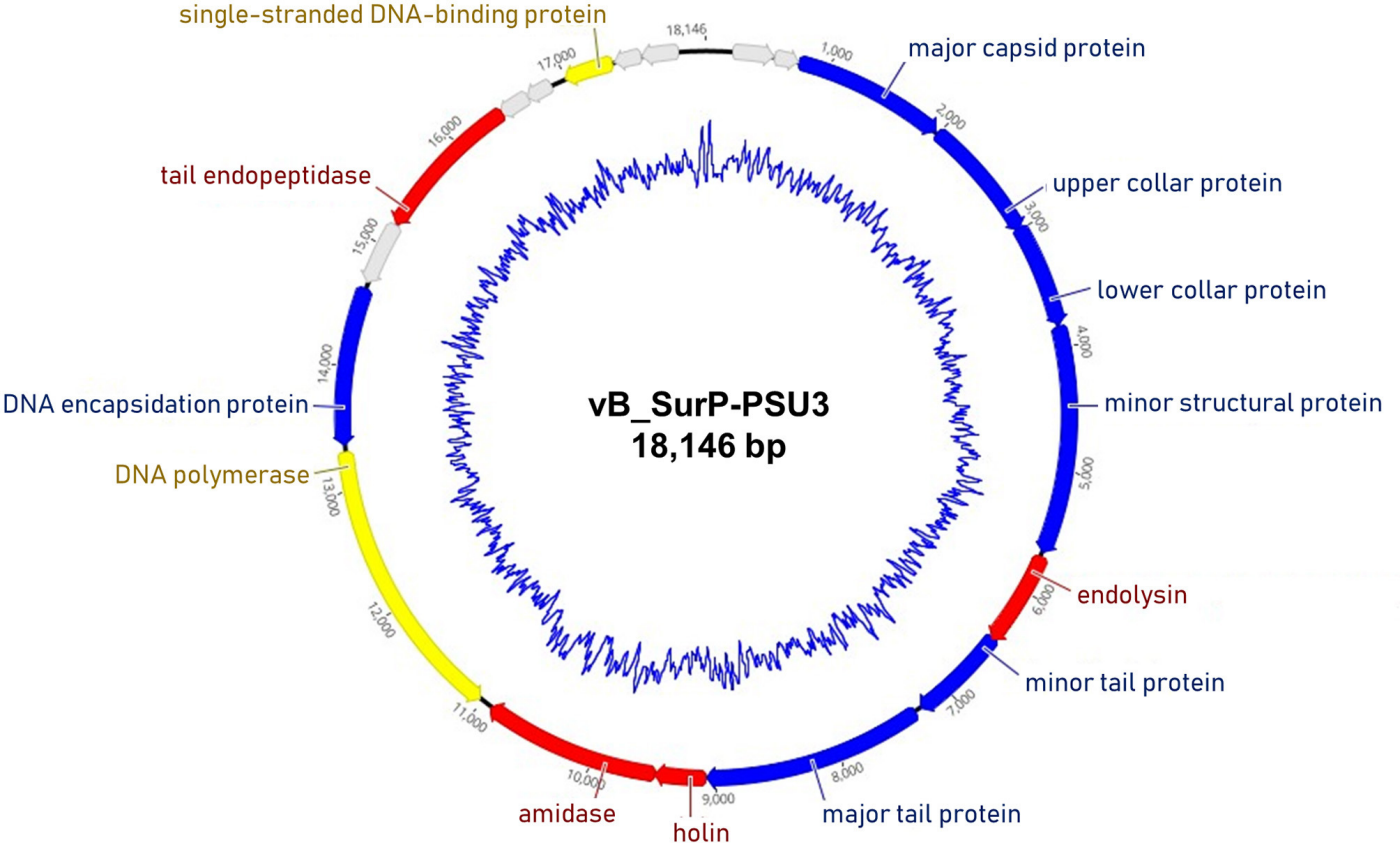
Whole-genome map of phage vB_SurP-PSU3. The arrow of each open reading frame (ORF) indicates the direction of the transcription. The predicted functions of ORFs are classified with different colors: nucleotide metabolism (yellow), structural and packaging (blue), lysis related (red), and hypothetical protein (gray). The inner blue circle indicates the G + C content.

**Figure 5:**
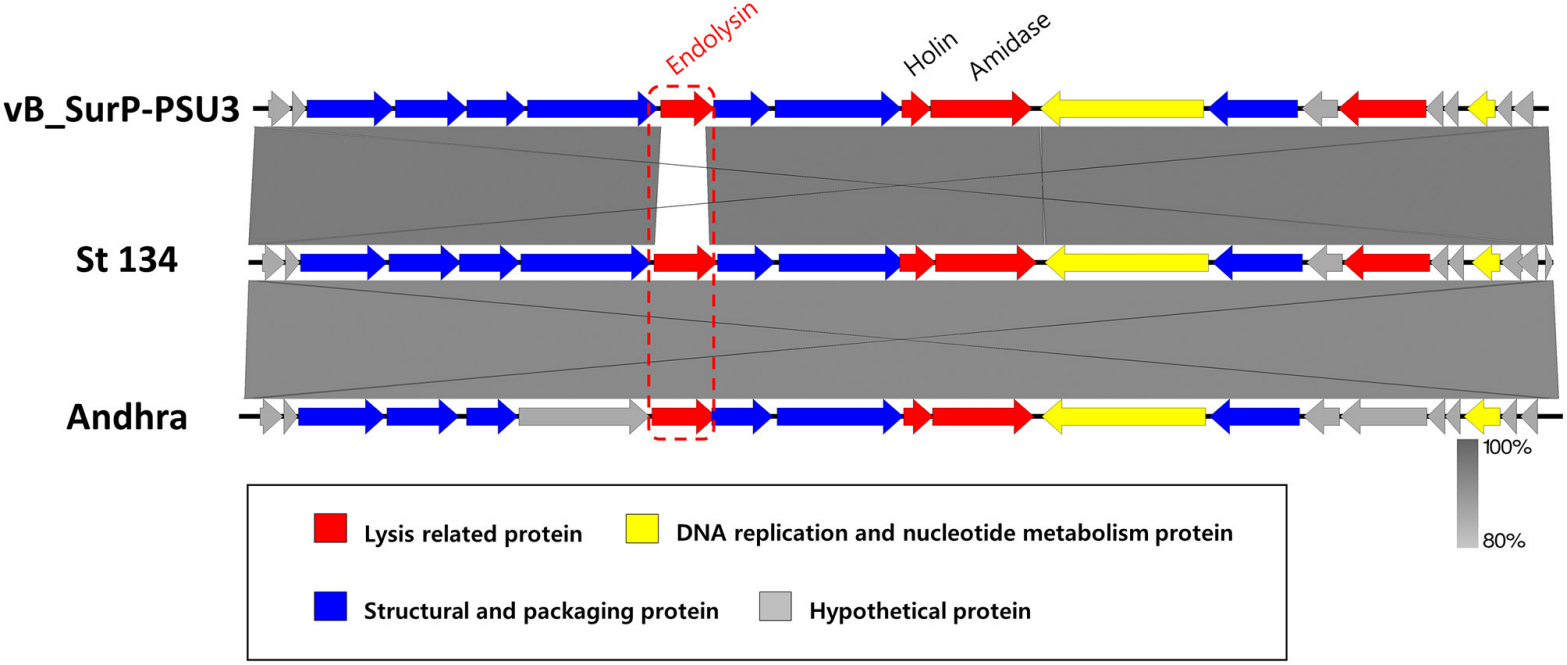
Comparative genomic analysis of phage vB_SurP-PSU3. Genome comparison of phage vB_SurP-PSU3 and the representative phages belonging to *Andhravirus*. The arrows indicate the direction of the transcriptional ORFs and the predicted functions of ORFs classified with different colors. The similarity between sequences was represented by darkness on the gray-scale bar.

**Figure 6:**
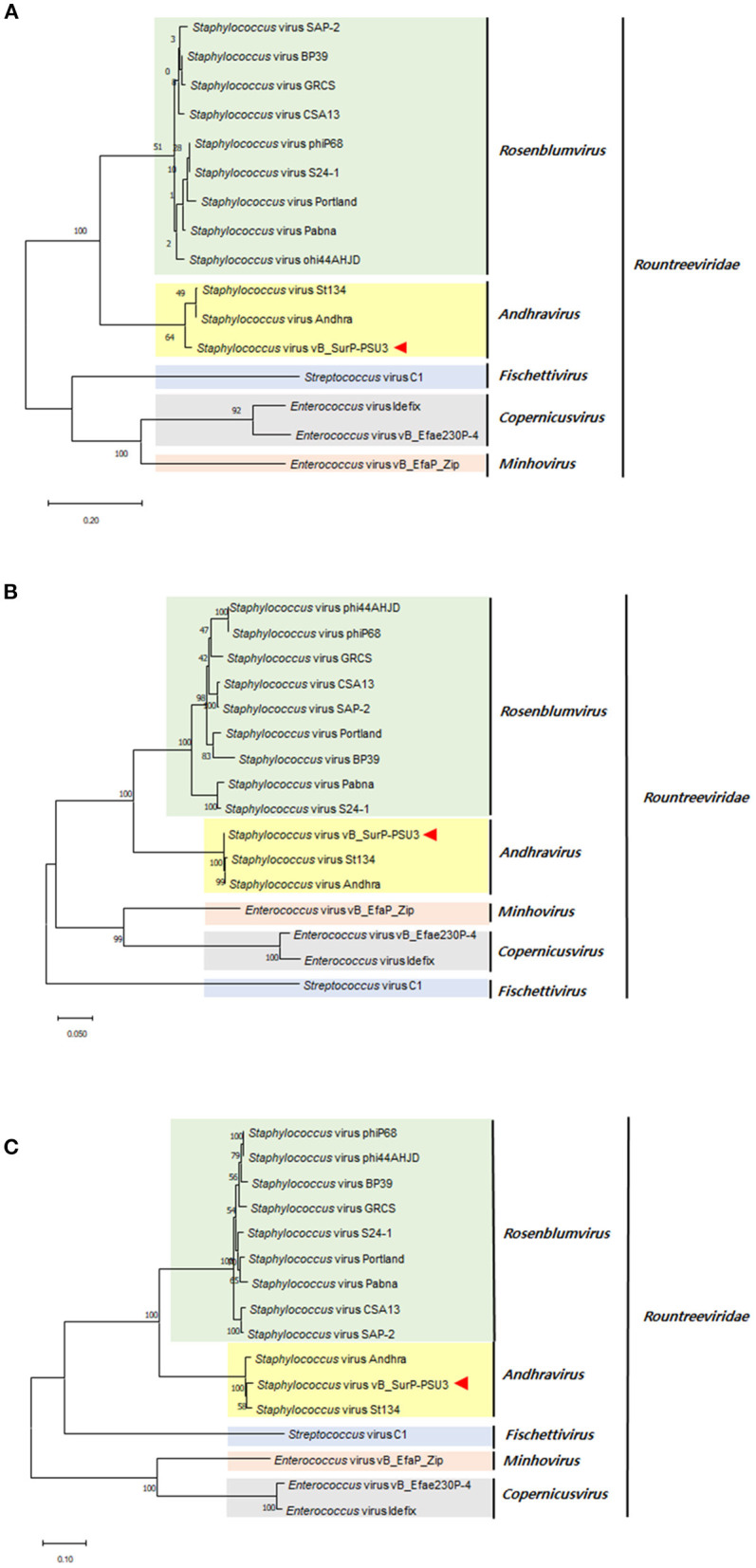
Phylogenetic trees of phage vB_SurP-PSU3. **(A)** Whole-genome. **(B)** Major capsid protein. **(C)** DNA polymerase.

The genome of phage vB_SurP-PSU3 was sequenced using the Illumina short-read NGS platform, and 3,829,468 reads were obtained with 211x coverage. The phage genome consisted of double-stranded linear DNA with 18,146 base pairs and a G + C content of 30.06% (Figure 4). Annotation analysis, based on the RAST program, revealed 20 ORFs, of which 11 ORFs (55%) were positive strands, and by BLASTp and HHpred, 13 ORFs (65%) had predicted functions classified into three functional groups: nucleotide metabolism, structural and packaging, and lysis-related (Supplementary Table S1). tRNA genes were not identified, and no virulence-or lysogeny-related genes were detected. BLASTn revealed that the genome of vB_SurP-PSU3 was most similar with *Staphylococcus* virus St134 (NC_047814, 95.17% identity and 96% coverage) and *Staphylococcus* virus *Andhra* (NC_047813, 93.44% identity and 95% coverage), both of which are representative species of the genus *Andhravirus* (Virus, 2011). Comparative genomic analysis between vB_SurP-PSU3, St134, and Andhra, showed that the ORFs of the three phages seemed highly similar, with the exception of ORF 7, which was translated into endolysin, one of the lysis-related proteins (Figure 5). Phylogenetic trees were constructed using the whole-genome (Figure 6A), major capsid protein (Figure 6B), and DNA polymerase (Figure 6C). As with the BLASTn results, all phylogenetic trees revealed that the phage belonged to the genus *Andhravirus* in the order *Caudovirales*, subfamily *Rakietenvirinae*, family *Rountreeviridae*. Based on these results, the newly isolated phage vB_SurP-PSU3 was classified as a new member of *Andhravirus*.

### Anti-biofilm Activity of Phage vB_SurP-PSU3

**Figure 7:**
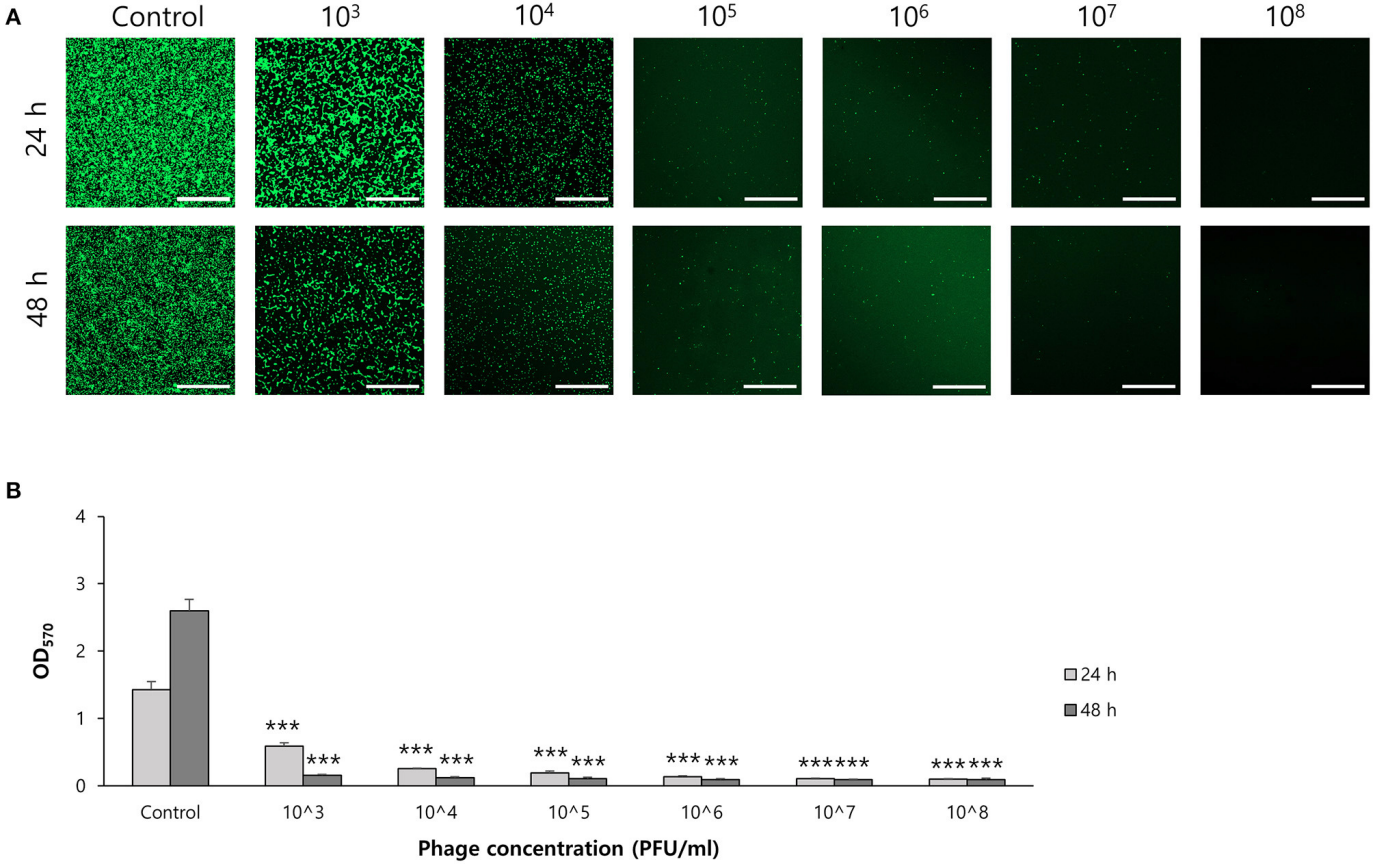
Biofilm prevention capability of phage vB_SurP-PSU3. **(A)** Confocal laser scanning microscopy of phage-treated bacterial cells in biofilm. Scale bar = 100 μm. **(B)** Stained biofilm with crystal violet at OD_570_. The data represents the mean ± SD of three independent experiments. An asterisk (***) indicates a significant difference between the control and experimental groups (P < 0.001).

**Figure 8:**
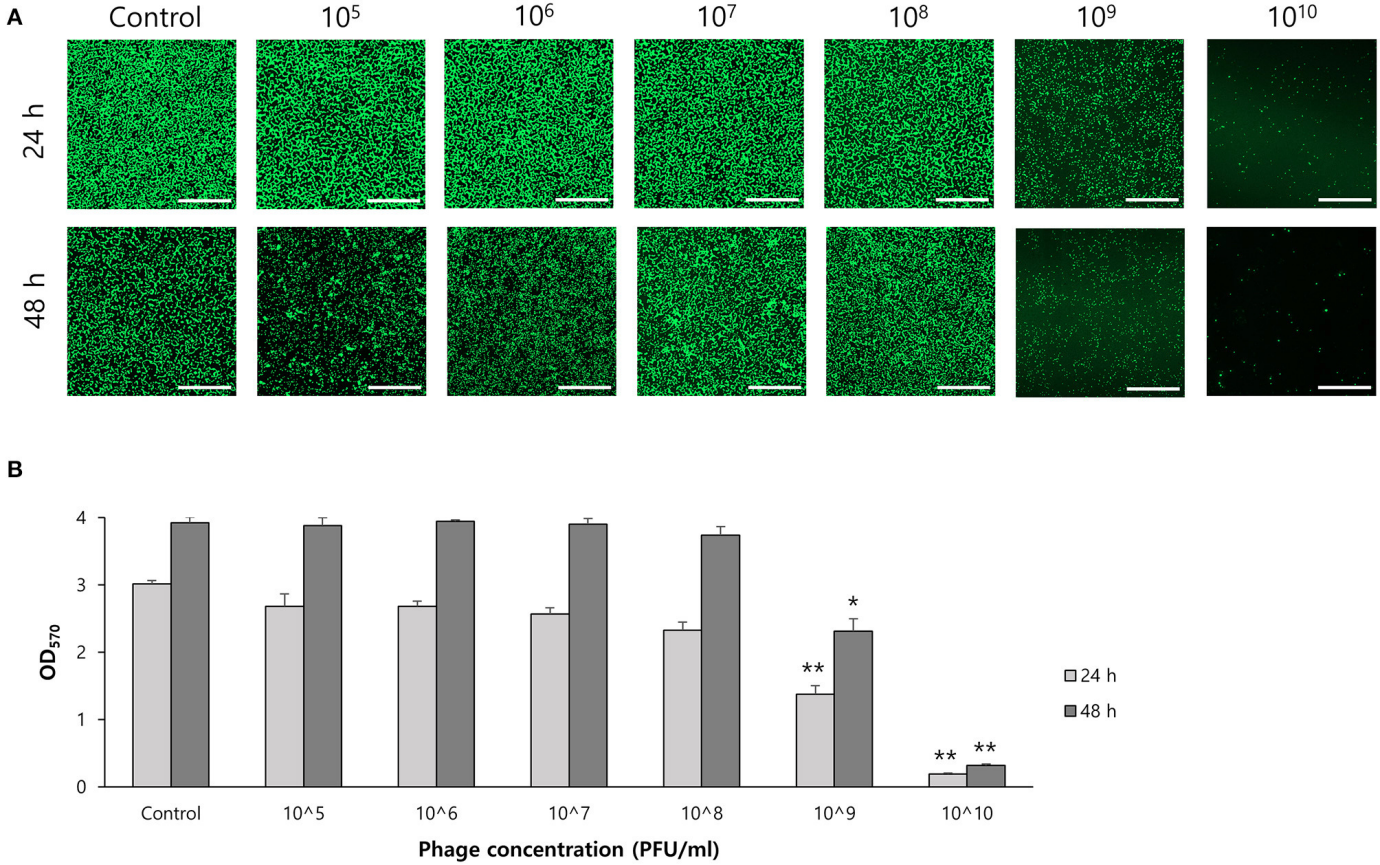
Biofilm degradation capability of phage vB_SurP-PSU3. **(A)** Confocal laser scanning microscopy of phage-treated bacterial cells in biofilm. Scale bar = 100 μm. **(B)** Stained biofilm with crystal violet at OD_570_. The data represents the mean ± SD of three independent experiments. Asterisks indicate a significant difference between the control and experimental groups: *P < 0.01; **P < 0.001.

To determine the effect of phage on biofilm formation, *S. ureilyticus* was incubated with different titers of phage suspension in 6-well polystyrene plates with coverslips and 96-well polystyrene plates (Figures 7A,B). Both results showed that biofilm formation was successfully prevented in the phage-treated wells compared to the control group. Even though the phage titer was low (10^3^ PFU/ml), the phage's capability to prevent biofilm formation was maintained until 48 h. For determining the effect of phages on biofilm degradation, biofilm was formed in 6-well polystyrene plates with coverslips and 96-well polystyrene plates, and each well was treated with different titers of phage suspension (Figures 8A,B). Similar to the biofilm prevention capability, the phage gradually decomposed the biofilm as the titer increased, but a relatively low dose of phage titer (up to 10^8^ PFU/ml) could not destroy the mature biofilm.

### Cell Cytotoxicity of Phage vB_SurP-PSU3

**Figure 9:**
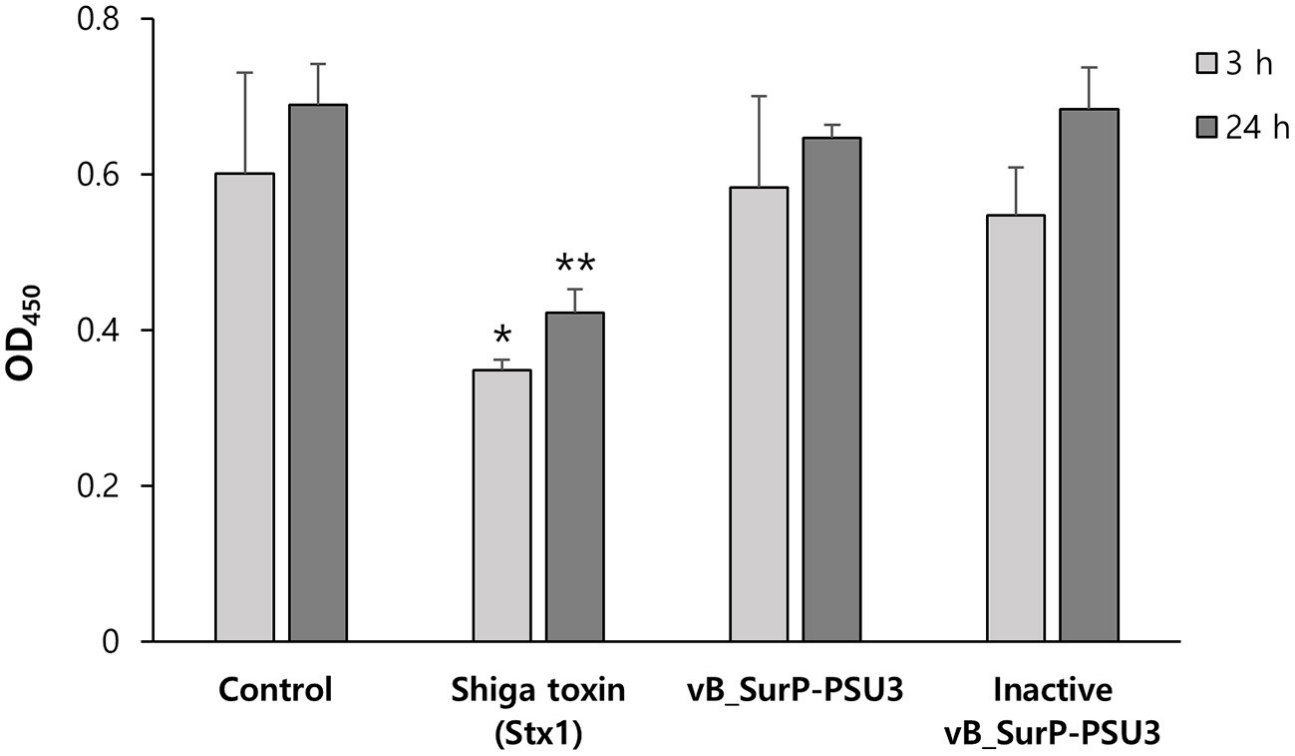
Cell cytotoxicity of phage vB_SurP-PSU3. The proliferation of bacterial cells against phage was determined using the WST assay at OD_450_. The data represents the mean ± SD of three independent experiments. Asterisks indicate significant differences: *P < 0.05; **P < 0.01.

The WST-8 assay, a cell proliferation assay, was conducted to evaluate the safety of the phage against mammalian cells (Figure 9). Vero cells (10^5^ cells/well) and activated or inactivated phage suspensions (10^5^ PFU/ml) were incubated together. There were no significant differences between the control group and activated or inactivated phage-treated groups. However, in the positive control treated with Shiga toxin (Stx1), cell viability decreased.

## Discussion

*Staphylococcus* can be classified into coagulase-negative staphylococci (CoNS) and coagulase-positive staphylococci (CoPS) based on the presence of the coagulase enzyme, a virulence factor that clots blood plasma. Among the genus *Staphylococcus, S. aureus*, a representative strain of CoPS, is considered the only major pathogen that causes diverse infectious diseases; however, as antibiotic resistance has arisen as a global concern, researchers have focused on the pathogenesis of CoNS. As commensal bacteria, CoNS are found commonly in daily lives, but nosocomial infections of CoNS in health facilities such as hospitals are increasing with the use of foreign bodies. Currently, CoNS are considered opportunistic pathogens that cause life-threatening diseases in immunocompromised patients (Wisplinghoff et al., 2004; Heilmann et al., 2019). Specifically, because biofilms of pathogens can protect microbial communities from external factors such as antibiotics, the biofilm-forming ability is a major pathogenic factor that can cause nosocomial infections and accounts for more than 80% of bacterial infections in humans (Jamal et al., 2018).

*S. ureilyticus*, one of the CoNS, originally belonged to *S. cohnii subsp. urealyticus* but was reclassified in 2020 (Madhaiyan et al., 2020). Many cases of antibiotic resistance have been reported in *S. ureilyticus*, and the frequency of human infections has increased (Soldera et al., 2013; Singh et al., 2016). Additionally, staphylococcal cassette chromosome mec (SCCmec), a mobile genetic element of staphylococcal species that encodes the mecA gene responsible for methicillin resistance, has been identified within genomes of clinical isolates (Zong and Lü, 2010; Mendoza-Olazarán et al., 2017). As SCCmec can be transferred to other staphylococcal strains, a novel strategy is needed to overcome antibiotic resistance in *S. ureilyticus* infections. As one of the most notable antibiotic alternatives, phages do not affect normal flora, and they can evolve their genetic information to ensure an effective infection of host bacteria. This indicates that phages can diminish resistance to treatment. Because of these advantages, multiple reports have demonstrated their ability to treat patients with bacterial pathogens (Kakasis and Panitsa, 2019; Düzgüneş et al., 2021); however, no phage has successfully infected and lysed *S. ureilyticus*.

In this study, phage vB_SurP-PSU3 was isolated from wastewater of a sewage treatment plant. The phage effectively lysed *S. ureilyticus* ATCC 49330 and some *S. warneri* strains (Table 1), which are responsible for coagulase-negative urinary tract infections (UTI) (Kanuparthy et al., 2020). *S. ureilyticus* and *S. warneri* are closely related to human daily lives as potential pathogens that require control. Genome-based molecular phylogenetic analysis revealed that phage vB_SurP-PSU3 belonged to *Andhravirus* and overall genomic contents and arrangements of the phage were similar to those of other previously reported members of *Andhravirus* (Virus, 2011). However, the phage vB_SurP-PSU3 showed several differences between the two phages in the *Andhravirus*: (i) Unlike vB_SurP-PSU3, St 134 and Andhra mainly infected *S. epidermidis*, and its possible infection of other species in CoNS (specifically *S. ureilyticus* or *S. warneri*) has not been reported (Cater et al., 2017). (ii) Although all three phages possessed a holin-endolysin-based lysis system and its predicted holin (ORF 10) in vB_SurP-PSU3 was almost identical to those of the other two phages, the predicted endolysin (ORF 7) in our isolate was distinctly different from St 134 (51.52% identity with 38% coverage) and Andhra (58.25% identity with 40% coverage) (Figure 5). The holin-endolysin lysis system is a programmed mechanism for lysing host cells during phage development. Specifically, endolysin, which is synonymous with phage lysins, is an enzyme that can degrade the peptidoglycan layer from inside the host bacteria. Because gram-positive bacteria such as *Staphylococcus* spp. do not have an outer membrane, phage endolysins can lyse them effectively (Schmelcher et al., 2012). In general, phage tail proteins interact with bacterial surface receptors and are responsible for determining potential host specificity (Le et al., 2013); however, the predicted tail proteins (ORF 8 and 9) in vB_SurP-PSU3 were very similar (99.15% identity with 100% coverage) to St 134 and Andhra (97.45% identity with 100% coverage), thereby suggesting that the difference in endolysin of our isolate may have contributed to the potential difference in host range, and further studies on the detailed characterization of its endolysin are now in progress.

Phage vB_SurP-PSU3 effectively reduced and removed staphylococcal biofilms. Previous research on phages has focused on their capacity to lyse planktonic bacteria. However, because of the importance of biofilms in antibiotic tolerance and resistance, new treatments are needed, and phages have been considered an interesting alternative (Ferriol-González and Domingo-Calap, 2020). Not all phages that demonstrate a lysis capability in planktonic cells are effective in biofilms; however, recent studies have reported that several staphylococcal phages can control bacterial biofilms (Cha et al., 2019; Kim et al., 2021). The isolated phage in this study, vB_SurP-PSU3, could successfully inhibit the growth of planktonic cells (Figure 3C), prevented the formation of *S. ureilyticus* biofilm, and degraded mature biofilm (Figures 7, 8). Although biofilm was not effectively degraded at titers below 10^8^ PFU/ml, high titers of phages eliminated mature biofilm for until 48 h. In the CLSM images of live *S. ureilyticus* in biofilm (Figures 7A, 8A), the mass of biofilm seemed to reduce at 48 h compared to 24 h; this suggests that *S. ureilyticus* ATCC 49330 may be able to degrade its own biofilm, as part of the lifecycle. Several previous studies have described biofilm dispersion within their lifecycle, and this process is assumed to be converted to the planktonic mode of growth because of steepening chemical concentration gradients, such as nutrient resources (Rumbaugh and Sauer, 2020).

Studies on considering phage as an antimicrobial agent have been conducted for decades. However, with the emergence of antibiotics, the development of phages has slowed down. Currently, the increasing number of AMR infections has drawn attention back to phages, and researchers have been investigating them to enhance their antibacterial activity for more effective use. Examples of good options include using a phage cocktail (Yu et al., 2022), a phage-antibiotic combination (Lin et al., 2021), and a phage-nanoparticle combination (Abdelsattar et al., 2021). Along with other antimicrobial agents, the remarkable effect of phage has been proven. Therefore, these biological approaches will suggest a way to treat a wide spectrum of bacterial pathogens more quickly.

In conclusion, the lytic phage vB_SurP-PSU3 was successfully isolated and characterized. The phage was effective in planktonic cells as well as in anti-biofilm, and cell cytotoxicity assays demonstrated its safety against potential applications in animals. Based on these results, vB_SurP-PSU3 is a promising biocontrol agent against *S. ureilyticus* and its biofilm. This phage is expected to have diverse applications, including prevention and treatment, which will be different from those of other reported phages. Therefore, further studies are necessary to reveal the function of hypothetical proteins for safer use. Moreover, identifying and engineering its lysin can be expected to be more effective than utilizing other antimicrobial agents.

## Data Availability Statement

The data presented in this study can be found in the GenBank database, accession number OK574338.

## Author Contributions

HK and SYP: conceptualization, methodology, conducted the study, data analysis, and writing the manuscript. M-SK: methodology, conducted the study, and edited the manuscript. SK and SCP: supplied materials for the study and editing of the manuscript. JK: conceptualization, funds, writing the manuscript, supervision, and editing the manuscript. All authors contributed to the article and approved the submitted version.

## Funding

This research was supported by the KRIBB Initiative Programs, the National Research Foundation of Korea (NRF-2020R1I1A2068827) funded by the Ministry of Education in the Republic of Korea, and also supported by the Development of technology for biomaterialization of marine fisheries by-products of Korea Institute of Marine Science & Technology Promotion (KIMST) funded by the Ministry of Oceans and Fisheries (KIMST-20220128).

## Conflict of Interest

The authors declare that the research was conducted in the absence of any commercial or financial relationships that could be construed as a potential conflict of interest.

## Publisher's Note

All claims expressed in this article are solely those of the authors and do not necessarily represent those of their affiliated organizations, or those of the publisher, the editors and the reviewers. Any product that may be evaluated in this article, or claim that may be made by its manufacturer, is not guaranteed or endorsed by the publisher.
