# Supplementary material for: Characterization of a Lytic Bacteriophage vB_SurP-PSU3 Infecting Staphylococcus ureilyticus and Its Efficacy Against Biofilm
Source: Front Microbiol. 2022 Jul 18;13:925866. doi: 10.3389/fmicb.2022.925866 (PMC9340203; doi:10.3389/fmicb.2022.925866)
Supplement: Supplementary file 1 [file Table_1.DOCX]

**Supplementary Table S1**. Predicted ORFs and their putative functions of vB_SurP-PSU3

| **Functional group** | **Gene** | | | **Gene product** | | | **Amino acid**  **Identity (%)** | **Putative function (E-value)** | **Conserved domain**  **[Family ID; Family; Description]** | **Predicted TMH**  **and signal peptide** | |  |
| --- | --- | --- | --- | --- | --- | --- | --- | --- | --- | --- | --- | --- |
|  | **ORF**  **No.** | **Range** | **Strand** | **aa size** | **MW**  **(kD)** | **pI** |  |  |  |  |  |  |
|  |  |  |  |  |  |  |  |  |  | **TMHHM** | **SignalP** | |
|  | 1 | 213-536 | + | 107 | 12294.2 | 3.96 | 100 | hypothetical protein  [*Staphylococcus* phage Andhra] (6e-65) |  | 0 | N | |
|  | 2 | 549-740 | + | 63 | 7160.7 | 4.92 | 98 | hypothetical protein  [*Staphylococcus* phage SeAlphi] (1e-37) |  | 0 | N | |
| structural and packaging | 3 | 753-1970 | + | 405 | 46362.4 | 5.04 | 99 | major capsid protein  [*Staphylococcus* phage Andhra] (0.0) |  | 0 | N | |
| structural and packaging | 4 | 1991-2998 | + | 335 | 39165.2 | 4.79 | 99 | upper collar protein  [*Staphylococcus* phage Andhra] (0.0) | [PF05352.15; Phage connector; Phage Connector (GP10)] | 0 | N | |
| structural and packaging | 5 | 2991-3827 | + | 278 | 33005.5 | 4.99 | 97 | lower collar protein  [*Staphylococcus* phage SeAlphi] (0.0) |  | 0 | N | |
| structural and packaging | 6 | 3840-5669 | + | 609 | 67305.4 | 5.64 | 99 | minor structural protein  [*Staphylococcus* phage Andhra] (0.0) |  | 0 | N | |
| lysis-related | 7 | 5706-6458 | + | 250 | 29455.4 | 9.30 | 48 | endolysin  [*Staphylococcus* phage S13’] (7e-70) | [PF05257.19; CHAP; CHAP domain] | 0 | N | |
| structural and packaging | 8 | 6448-7251 | + | 267 | 30514.4 | 5.08 | 97 | minor tail protein  [*Staphylococcus* phage vB_SepP_BE03] (0.0) |  | 0 | N | |
| structural and packaging | 9 | 7311-9077 | + | 588 | 68402.2 | 5.88 | 99 | major tail protein  [*Staphylococcus* phage vB_SepP_BE03] (0.0) | [PF16838.8; Caud_tail_N; Caudoviral major tail protein N-terminus] | 0 | N | |
| lysis-related | 10 | 9086-9496 | + | 136 | 15445.4 | 6.57 | 99 | holin  [*Staphylococcus* phage JBug18] (1e-91) | [PF05105.15; Phage_holin_4_1; Bacteriophage holin family] | 2 | N | |
| lysis-related | 11 | 9489-10910 | + | 473 | 51534.6 | 8.97 | 99 | amidase  [*Staphylococcus* phage vB_SepP_BE03] (0.0) |  | 0 | N | |
| DNA replication and  nucleotide metabolism | 12 | 11022-13313 | - | 763 | 90476.5 | 5.62 | 97 | DNA polymerase  [*Staphylococcus* phage Pike] (0.0) |  | 0 | N | |
| DNA replication and  nucleotide metabolism | 13 | 13371-14630 | - | 419 | 50100.6 | 6.61 | 99 | DNA encapsidation protein  [*Staphylococcus* phage SeAlphi] (0.0) |  | 0 | N | |
|  | 14 | 14689-15192 | - | 167 | 19831.4 | 9.25 | 99 | hypothetical protein  [*Staphylococcus* phage St134] (5e-117) |  | 0 | N | |
| lysis-related | 15 | 15203-16432 | - | 409 | 46980.8 | 6.06 | 95 | tail endopeptidase  [*Staphylococcus* phage SeAlphi] (0.0) | [PF13472.9; Lipase_GDSL_2; GDSL-like Lipase/Acylhydrolase family] | 0 | N | |
|  | 16 | 16422-16667 | - | 81 | 9399.8 | 4.72 | 100 | hypothetical protein  [*Staphylococcus* phage Andhra] (4e-50) |  | 1 | N | |
|  | 17 | 16671-16880 | - | 69 | 7961.3 | 7.71 | 96 | hypothetical protein  [*Staphylococcus* phage Pike] (6e-39) |  | 0 | N | |
| DNA replication and  nucleotide metabolism | 18 | 17006-17398 | - | 130 | 15122.0 | 5.03 | 94 | single stranded DNA-binding protein  [*Staphylococcus* phage SeAlphi] (1e-83) |  | 0 | N | |
|  | 19 | 17413-17628 | - | 71 | 8607.9 | 6.27 | 100 | hypothetical protein  [*Staphylococcus* phage vB_SepP_BE03] (1e-43) |  | 0 | N | |
|  | 20 | 17645-17932 | - | 95 | 11226.6 | 4.42 | 97 | hypothetical protein  [*Staphylococcus* phage JBug18] (2e-61) |  | 0 | N | |
